# The Associations of Serum Serotonin with Bone Traits Are Age- and Gender-Specific

**DOI:** 10.1371/journal.pone.0109028

**Published:** 2014-10-03

**Authors:** Qin Wang, Decai Chen, Patrick Nicholson, Shumei Cheng, Markku Alen, Lijian Mao, Sulin Cheng

**Affiliations:** 1 Department of Endocrinology, West China Hospital of Sichuan University, Sichuan, China; 2 Department of Health Sciences, University of Jyväskylä, Jyväskylä, Finland; 3 Department of Medical Rehabilitation, Oulu University Hospital and Institute of Health Sciences, University of Oulu, Oulu, Finland; 4 Department of Sport and Physical Education, Shanghai Jiao Tong University, Shanghai, China; Université Jean Monnet, France

## Abstract

**Context:**

Serotonin plays a potential role in bone metabolism, but the nature and extent of this relationship is unclear and human studies directly addressing the skeletal effect of circulating serotonin are rare.

**Objective:**

The study aimed to investigate the associations between serum serotonin and bone traits at multiple skeletal sites in women and men.

**Subjects and Methods:**

Subjects were part of the CALEX-family study and comprised 235 young women, 121 premenopausal women, 124 postmenopausal women, and 168 men. Body composition was assessed using DXA, as was areal bone mineral density (aBMD) of spine, femur and whole body. In addition, pQCT was used to determine bone properties at tibial midshaft and distal radius. Fasting serum serotonin concentration was assessed using a competitive enzyme-linked immunosorbent assay.

**Results:**

Serum serotonin declined with advancing age both in females and males (all p<0.01). Serotonin was negatively correlated with weight, BMI, lean and fat mass in women (r = −0.22 to −0.39, all p<0.001), but positively with height and lean mass in men (all p<0.01). In the premenopausal women, serotonin was negatively correlated with lumbar spine aBMD (*r* = −0.23, p<0.05) but the statistical significance disappeared after adjustment for weight. Conversely, in postmenopausal women, serotonin was positively correlated with whole body and femur aBMD, as well as with distal radius bone mineral content and volumetric BMD (*r* = 0.20 to 0.30, all p<0.05), and these associations remained significant after adjustment for weight. In men, no significant associations were found between serotonin and bone traits.

**Conclusion:**

Serum serotonin is positively associated with bone traits in postmenopausal women, but not in premenopausal women or men. This partially supports the idea of circulating serotonin playing a role in the regulation of bone metabolism, but also indicates the importance of gender and age specific factors.

## Introduction

Serotonin (5-hydroxytryptamine, 5-HT) is a neuropeptide that has received intensive attention recently due to its potential role in bone metabolism [1], [2]. Previous human studies showed increased hip and wrist fractures [3]–[5] and decreased bone mineral density (BMD) in femoral neck and total hip in patients with depression treated by selective serotonin reuptake inhibitors (SSRIs) [4], [6], [7]. The combination of compromised bone and low serum serotonin concentration in SSRIs-users [8]–[10] apparently contradicts the notion of an inhibitory effect of circulating serotonin on bone formation implied by recent animal studies [1], [2]. This suggests that the skeletal effects of circulating serotonin may not be as straightforward as currently portrayed. However, human studies directly addressing the skeletal effect of circulating serotonin are rare.

In order to provide further data on serotonin and bone in healthy subjects, we investigated associations between serum serotonin and bone traits at multiple sites in Finnish women spanning a wide age range, as well as in men for whom no data have yet been reported in the literature.

## Subjects and Methods

### Subjects

The study population of this report is a part of the CALEX-family study that has been described elsewhere [11]–[13]. Briefly, 235 Finnish young women (mean age 18.3 yrs, range 16.1–21.4 yrs) participated in the laboratory tests in the year 2007–8. In addition, 40 brothers, 214 mothers, 121 fathers, 120 grandmothers and 65 grandfathers of these girls were also invited to participate in the measurements. In these family members, 89 adult women and 58 men were excluded due to having diseases or medications (including HRT) that affect bone metabolism or serum serotonin level (i.e. SSRIs, adrenergic blockers, adrenergic stimulants, alpha blockers, beta blockers, alpha-adrenergic agonists, anticholinergic agents, antidepressants, antipsychotics, anticonvulsants, and gastrointestinal prokinetic agents, corticosteroids, anti-osteoporotic drugs). Finally, in addition to the young women already mentioned, 121 premenopausal (mean age 46.1 yrs, range 38.0–54.3 yrs) women, 124 postmenopausal women (66.5 yrs, 45.5–80.3 yrs), 112 men under 60 yrs old (50.0 yrs, 41.7–59.5 yrs) and 56 men over 60 yrs old (72.9 yrs, 61.6–83.8 yrs) were eligible and included in present study.

The study protocol was approved by the ethical committee of the Central Finland Health Care District. Informed consent was given by all subjects prior to the assessments.

### Methods

#### Body composition and bone measurements

Body composition and bone traits were assessed using dual-energy X-ray absorptiometry (DXA Prodigy; GE Lunar Corp., Madison, WI, USA). Total body weight (TW, kg) was the sum of bone mass (BM, kg), lean mass (LM, kg), and fat mass (FM, kg). Bone traits included bone mineral content (BMC), areal bone mineral density (aBMD) and bone area (BA) of the whole body (WB), total femur (TF), femoral neck (FN) and lumbar spine (L_2_–L_4_). The coefficient of variation (CV) of repeated measurements ranged from 0.6% to 1.2% for BMC, from 0.9% to 1.3% for BMD, and from 0.6% to 1.2% for BA.

The left tibial and radial shafts were scanned using peripheral quantitative computerized tomography (XCT 2000; Stratec Medizintechnik, Pforzheim, Germany). The scan locations were at 60% of lower leg length up from the lateral malleolus and 4% of forearm length proximal to the wrist joint surface. The bone parameters included bone mineral content (BMC, mg/mm), bone cross-sectional area (CSA, mm^2^) and volumetric bone mineral density (vBMD, mg/cm^3^). The coefficient of variation (CV) of two repeated measurements on the same subject on the same day was on average 1% for total CSA, BMC, and <1% for vBMD.

#### Blood sample collection and measurements

Fasting blood samples were collected in the morning between 7 am and 9 am. The subjects ate according to their habitual diet without any restrictions on the day before blood sampling. In premenopausal women, blood was taken between 2 and 5 days after onset of menstruation. Serum was separated and stored immediately in aliquot at −80°C until analyzed. All samples were analyzed by one technician using the same kits and instruments.

Serum serotonin concentration was measured using a competitive enzyme-linked immunosorbent assay (ELISA; Immuno-Biological Laboratories, GmbH., Hamburg, German). The inter-assay and intra-assay CV were 3.8% and 3.7% respectively. Serum total osteocalcin (OC) was determined by previously described two-site immunoassay [14], and the intra- and inter- assay CV were less than 5% and 8%, respectively.

### Statistical analysis

All data were checked for normality using the Shapiro-Wilk's W-test in SPSS 15.0 for Windows. For data that were not normally distributed, the natural logarithm was used. Analysis of variance with Least Significance Deviation post-hoc test was used to test the differences among groups. Correlations were evaluated using Pearson correlation coefficients or partial correlation coefficients. Differences were considered significant if p<0.05.

## Results

Young and premenopausal women had higher BMC and BMD than postmenopausal women at all skeletal sites (all p<0.05, Table 1). In comparison with their younger counterparts, men above 60 yrs old had significantly lower aBMD of the WB, TF and FN, lower BMC and vBMD of the tibial shaft and distal radius (all p<0.05, Table 2).

**Table 1:** Anthropometric, body composition, serum serotonin, sex hormones and bone parameters in women.

	All Women	Young Adult Women	Premenopausal Adult Women	Postmenopausal Adult Women
**N**	480	235	121	124
**Age (yr)**	38.0±21.1	18.3±1.1	46.1±3.6^ab^	66.5±10.1^c^
**Height(cm)**	164.4±6.2	165.8±5.7	165.4±6.0^b^	160.7±5.8^c^
**Weight(kg)**	64.9±12.0	60.1±9.6	69.1±12.7^a^	69.7±12.0^c^
**BMI(kg/m^2^)**	24.1±4.6	21.9±3.1	25.3±4.4^ab^	27.1±4.8^c^
**Lean Mass(kg)**	39.6±4.4	38.1±4.2	41.2±4.7^ab^	39.6±4.4^c^
**Fat Mass(kg)**	23.0±9.5	19.3±7.5	25.1±10.0^ab^	27.9±9.6^c^
**Serotonin (ng/ml)**	205±74	238±63	187±70^ab^	165±70^c^
**Osteocalcin (ng/ml)**	8.37(5.82, 11.67)	10.81(8.41, 13.28)	5.25(4.27, 7.15)^ab^	7.05(5.47, 10.07)^c^
***DXA-aBMD(g/cm^2^)***				
** Total body**	1.17±0.10	1.16±0.07	1.23±0.09^ab^	1.12±0.11^c^
** Total Femur**	1.03±0.14	1.07±0.13	1.05±0.12^b^	0.94±0.15^c^
** Femoral Neck**	0.99±0.15	1.06±0.14	0.99±0.12^ab^	0.87±0.14^c^
** Total Spine**	1.05±0.12	1.03±0.10	1.11±0.13^ab^	1.01±0.14
** Lumbar Spine**	1.20±0.15	1.20±0.12	1.27±0.15^ab^	1.14±0.18^c^
***pQCT-Tibial shaft***				
** BMC (g/mm)**	358±46	355±43	380±46^ab^	342±45
** vBMD(mg/cm^3^)**	738±72	753±51	766±59^b^	682±83^c^
** CSA(mm^2^)**	486±59	471±57	497±62^a^	503±53^c^
** cBMC (g/mm)**	312±46	311±40	334±43^ab^	291±49^c^
** cBMD(mg/cm^3^)**	1131±34	1135±21	1154±21^ab^	1102±43^c^
** cCSA(mm^2^)**	276±38	274±35	290±37^ab^	263±39^c^
***pQCT-Distal Radius***				
** BMC (g/mm)**	107±17	106±13	114±18^ab^	100±19^c^
** vBMD(mg/cm^3^)**	330±55	343±42	346±51^b^	289±58^c^
** CSA(mm^2^)**	328±51	312±41	332±48^ab^	353±59^c^
** tBMC (g/mm)**	27.1±6.5	27.6±5.4	27.3±7.3	25.8±7.4^c^
** tBMD(mg/cm^3^)**	185±40	198±34	183.3±40^ab^	164±42^c^
** tCSA(mm^2^)**	147±23	140±19	149±21^ab^	159±26^c^

Data are represented as mean±SD or median and quartile range (P_25_, P_75_). BMI: Bone mass index; BMC: bone mineral content; aBMD: areal bone mineral density; vBMD: volumetric bone mineral density; CSA: cross-sectional area. cBMC: cortical BMC; cBMD: cortical BMD; cCSA: cortical CSA; tBMC: trabecular BMC; tBMD: trabecular BMD; tCSA: trabecular CSA. Bone traits of total body, femur and spine were attained by DXA while those of tibia and radius were assessed by pQCT.

a : P<0.05 Premenopausal adult women vs. young adult women.

b : P<0.05 Premenopausal adult women vs. postmenopausal women.

c : P<0.05 Young adult women vs. postmenopausal women.

**Table 2:** Anthropometric, serum serotonin and bone parameters in men.

	All Men	Men aged <60-yr	Men aged ≥60-yr
**N**	168	112	56
**Age (yr)**	57.7±11.7	50.0±4.0	72.9±5.2^c^
**Height(cm)**	175.8±6.9	177.7±6.2	171.2±5.3^c^
**Weight(kg)**	82.1±10.6	83.6±10.0	79.1±10.6^a^
**BMI(kg/m^2^)**	26.6±3.2	26.5±3.0	26.8±3.4
**Lean Mass(kg)**	56.5±6.0	58.1±5.8	53.3±5.1^c^
**Fat Mass(kg)**	22.5±7.2	22.1±6.6	23.2±8.2
**Serotonin (ng/ml)**	173.9±59.3	186.6±59.2	146.9±59.3^c^
**Osteocalcin (ng/ml)**	6.75(5.57, 8.71)	7.18(5.91, 8.72)	6.14(5.28, 8.46)
***DXA-aBMD(g/cm^2^)***			
** Total Body**	1.27±0.10	1.28±0.10	1.24±0.11^b^
** Total Femur**	1.09±0.13	1.04±0.14	1.07±0.14^a^
** Femoral Neck**	0.98±0.13	1.01±0.13	0.93±0.13^b^
** Total Spine**	1.12±0.14	1.12±0.14	1.13±0.15
** Lumbar Spine**	1.24±0.20	1.22±0.20	1.27±0.21
***pQCT-Tibial shaft***			
** BMC (g/mm)**	465±59	473±59	447±55^b^
** BMD(mg/cm^3^)**	748±70	752±65	739±78^a^
** CSA(mm^2^)**	626±68	629±68	620±69
** cBMC (g/mm)**	410±57	418±55	392±60^b^
** cBMD(mg/cm^3^)**	1129±27	1131±25	1123±29^a^
** cCSA(mm^2^)**	362±47	369±45	348±48^b^
***pQCT-Distal Radius***			
** BMC (g/mm)**	161±25	163±23	157±29
** BMD(mg/cm^3^)**	375±57	389±48	344±64^c^
** CSA(mm^2^)**	434±65	422±58	460±70^c^
** tBMC (g/mm)**	42.2±8.7	41.6±9.1	42.2±8.7
** tBMD(mg/cm^3^)**	218±43	225±41	203±44^b^
** tCSA(mm^2^)**	195±29	190±26	207±32^c^

Data are represented as mean±SD or median and quartile range(P_25_, P_75_). BMI: Bone mass index; BMC: bone mineral content; aBMD: areal bone mineral density; vBMD: volumetric bone mineral density; CSA: cross-sectional area. cBMC: cortical BMC; cBMD: cortical BMD; cCSA: cortical CSA; tBMC: trabecular BMC; tBMD: trabecular BMD; tCSA: trabecular CSA. Bone traits of total body, femur and spine were attained by DXA while those of tibia and radius were from pQCT.

a : P<0.05; ^b^: P<0.01; ^c^: P<0.001 Men aged 18 to 60-yr versus Men aged ≥60-yr.

Serum serotonin level was negatively correlated with age in both women and men (*r* = −0.44 and −0.33, respectively, all p<0.001, **Table 3**). In women, serum serotonin was negatively correlated with weight, BMI, lean and fat mass (*r* = −0.22 to −0.39, all p<0.001). However, in men, the correlations of serotonin with height and lean mass were positive (all p<0.01). Young women had the highest level of osteocalcin, followed by the post- and premenopausal women (all p<0.01). Osteocalcin was positively correlated with serotonin only in the young women.

**Table 3:** Correlations between serum serotonin and age, anthropometric parameters, body composition and osteocalcin.

	Serum Serotonin						
	All Women	Young Women	Premenopausal Women	Postmenopausal Women	All Men	Men aged <60-yr	Men aged ≥60-yr
**N**	477	232	121	124	168	112	56
**Age (yr)**	**−0.44^c^**	−0.12	−0.17	−0.13	**−0.33^c^**	−0.17	<0.001
**Height(cm)**	**0.14^b^**	−0.06	−0.02	0.17	**0.26^c^**	−0.08	**0.41^c^**
**Weight(kg)**	**−0.34^c^**	**−0.15^a^**	**−0.26^b^**	**−0.22^a^**	0.10	0.09	−0.06
**BMI(kg/m^2^)**	**−0.41^c^**	**−0.15^a^**	**−0.27^b^**	**−0.32^c^**	−0.06	0.04	−0.22
**Lean Mass(kg)**	**−0.22^c^**	−0.13	−0.17	−0.10	**0.23^b^**	0.14	0.11
**Fat Mass(kg)**	**−0.39^c^**	−0.13	**−0.31^b^**	**−0.35^c^**	−0.04	0.05	−0.16
**Osteocalcin(ng/ml)**	**0.28^c^**	**0.18^b^**	−0.07	0.11	−0.05	−0.05	−0.12

Data presented are Pearson correlation coefficients.

a : P<0.05; ^b^: P<0.01; ^c^: P<0.001.

In premenopausal women, a negative correlation between serotonin level and aBMD at L_2_–L_4_ was found (*r* = −0.23, p<0.05), but the statistical significance disappeared after adjustment for weight (**Table 4**). No correlation was found between serotonin level and aBMD at L_2_–L_4_ in young women.

**Table 4:** Correlations between serum serotonin and bone traits.

	Serum Serotonin				
	Young Women	Premenopausal Women	Postmenopausal Women	Men aged <60-yr	Men aged ≥60-yr
N	232	121	124	112	56
***DXA-aBMD(g/cm^2^)***					
Whole Body	−0.13/−0.02	−0.14/0.13	**0.20^a^/0.34^c^**	−0.013/−0.12	0.26/0.25
Total Femur	−0.10/−0.02	−0.18/0.10	**0.26^b^/0.40^c^**	−0.07/−0.01	0.25/0.27
Femoral Neck	−0.06/0.03	−0.13/0.14	**0.30^b^/0.42^c^**	−0.10/−0.04	0.22/0.20
L_2_–L_4_	−0.12/−0.06	**−0.23^a^**/−0.01	0.09/**0.22^a^**	−0.13/−0.11	0.09/0.10
***pQCT-Tibial shaft***					
Total BMC (g/mm)	−0.13/−0.02	−0.12/−0.03	0.13/**0.30^b^**	−0.05/−0.15	0.13/0.01
Total vBMD(mg/cm^3^)	−0.05/−0.06	−0.01/0.12	0.15/**0.22^a^**	0.01/0.01	0.15/0.14
Total CSA(mm^2^)	−0.07/−0.03	−0.09/−0.12	−0.01/0.09	0.06/−0.05	0.21/0.09
Cortical BMC (g/mm)	−0.13/−0.02	−0.08/−0.002	0.14/**0.29^a^**	0.08/−0.01	0.24/0.20
Cortical vBMD(mg/cm^3^)	0.02/0.002	0.13/0.16	0.10/0.15	0.08/0.13	0.18/0.24
Cortical CSA(mm^2^)	−0.12/−0.02	−0.10/−0.03	0.15/**0.30^a^**	0.07/−0.02	0.26/0.18
***pQCT-Distal Radius***					
Total BMC (g/mm)	−0.10/−0.003	−0.09/0.09	**0.20^a^/0.35^c^**	−0.01/−0.16	0.20/0.20
Total vBMD(mg/cm^3^)	0.02/0.02	−0.02/0.13	**0.20^a^/0.32^b^**	0.05/0.005	0.06/0.15
Total CSA(mm^2^)	−0.10/−0.03	−0.07/−0.03	−0.03/−0.04	−0.03/−0.17	0.18/0.07
Trabecular BMC(g/mm)	−0.08/−0.004	−0.11/−0.04	0.16/**0.27^b^**	−0.13/−0.09	0.21/0.17
Trabecular vBMD(mg/cm^3^)	−0.01/0.01	−0.09/0.07	0.18/**0.29^b^**	−0.11/0.01	0.17/0.11
Trabecular CSA(mm^2^)	−0.11/−0.07	−0.07/−0.03	−0.03/−0.01	−0.03/−0.17	0.19/0.07

Data shown are unadjusted values of Pearson correlation coefficients/weight-adjusted partial correlation coefficients in women, and unadjusted values/height-adjusted values in men. BMI: body mass index; BMC: bone mineral content; aBMD: areal bone mineral density; vBMD: volumetric bone mineral density; CSA: cross-sectional area.

a : P<0.05; ^b^: P<0.01; ^c^: P<0.001.

Conversely, serotonin level was positively correlated with aBMD of WB, TF and FN, as well as total BMC and vBMD of the distal radius in postmenopausal women (*r* = 0.20 to 0.30, all p<0.05), with the strongest correlations found at FN and TF (*r* = 0.40 and 0.42, respectively, all p<0.001). After adjustment for weight, the positive correlations became even more pronounced (*r* = 0.22 to 0.42, all p<0.05) (**Table 4**).

In men, no significant associations were found between serotonin and any of the bone parameters (all p>0.05) (**Table 4**).

## Discussion

In this cross-sectional study, serum serotonin was positively associated with certain bone parameters in postmenopausal women, but not in premenopausal women or men. This finding accords with some previous studies [3]–[7]but contradicts others [1], [2].

The positive association between bone traits and serum serotonin in postmenopausal women suggests that higher levels of serum serotonin may be beneficial for bone, and lower levels detrimental. The latter inference is supported by the observation that SSRI-users have low serum serotonin level and compromised bone properties [8]–[10]. Several preclinical studies also tend to support this view. For example, serotonin has been shown to enhance the proliferation of primary human osteoblasts by increasing the release of prostaglandin-E_2_ [15]–[17]. Mice with a null mutation in the gene encoding for the 5-HT_2B_ receptor manifest reduced aBMD in whole body and femur, altered trabecular architecture in the tibia, as well as inferior mechanical properties [18]. However, the inhibitory effects of circulating serotonin on bone formation found in recent animal studies appear to contradict the notion of a positive role for serotonin in bone metabolism. Yadav and collegues demonstrated that circulating serotonin inhibits bone formation without affecting bone resorption [2]. Inhibition of tryptophan hydroxylase-1 (TPH-1), the rate-limiting enzyme in biosynthesis of circulating serotonin, leads to increased bone formation in ovariectomized rodents with osteoporosis [1]. In addition, a high bone mass phenotype with low circulating serotonin in *Lrp5*-mutated patients is also consistent with a detrimental skeletal effect of serotonin [1], [2], [19].

The discrepancy between studies makes interpretation of the role of circulating serotonin in bone metabolism difficult. Thus more evidence from population-based studies is called for. The only clinical study performed in normal human subjects, by Mödder and colleagues [20], does not provide convincing support to any side, even though they reported a trend for a negative association between serum serotonin and certain bone parameters in postmenopausal women. The correlations found in their study were not only weak, but also disappeared after adjusting for BMI or weight, which was a major confounding factor for the relationship of serotonin and bone. Serotonin affects body weight through the central regulation of appetite and food intake [21]–[24], while body weight is positively related to bone traits due to mechanical loading effects [25], [26]. Accordingly, we adjusted for weight in the analysis of the bone-serotonin correlations, and found that the positive associations in postmenopausal women became significant or even more pronounced, suggesting that the positive associations found in this study are robust. The statistical significance was similar after adjusting for BMI instead of body weight.

The inconsistency between Mödder et al's report and ours is probably due to cohort effects [20]. The average level of serum serotonin in the Finnish participants of this study was higher than that in Mödder et al's study which used the same assay. In addition, the different dietary habits between the two study populations may contribute to the discrepancy, since serum serotonin level is largely affected by tryptophan intake which may significantly differ between the populations. Except for the discrepancy, both studies showed the statistical significance only appeared in postmenopausal women, suggesting that the age or hormone status may contribute the mechanism behind the association between circulation serotonin and bone. Therefore, we also tested the relationships between sex hormones (estradiol and testosterone) and serotonin in all subjects but no significance was found (data not shown).

In conclusion, serum serotonin may play a positive role in bone metabolism in postmenopausal women, but not in premenopausal women or men. The effects of serotonin on bone are probably gender- and age-dependent. However, the mechanism of skeletal regulation by circulating serotonin in human body still remains elusive and further studies aimed at revealing the potential role of circulating serotonin in bone metabolism in humans are needed.

## References

[1] Yadav VK, Balaji S, Suresh PS, Liu XS, Lu X, et al (2010) Pharmacological inhibition of gut-derived serotonin synthesis is a potential bone anabolic treatment for osteoporosis. Nat Med 16: 308–312. 20139991 10.1038/nm.2098 PMC2836724

[2] Yadav VK, Ryu JH, Suda N, Tanaka KF, Gingrich JA, et al (2008) Lrp5 controls bone formation by inhibiting serotonin synthesis in the duodenum. Cell 135: 825–837. 19041748 10.1016/j.cell.2008.09.059 PMC2614332

[3] Liu B, Anderson G, Mittmann N, To T, Axcell T, et al (1998) Use of selective serotonin-reuptake inhibitors or tricyclic antidepressants and risk of hip fractures in elderly people. Lancet 351: 1303–1307. 9643791 10.1016/s0140-6736(97)09528-7

[4] Richards JB, Papaioannou A, Adachi JD, Joseph L, Whitson HE, et al (2007) Effect of selective serotonin reuptake inhibitors on the risk of fracture. Arch Intern Med 167: 188–194. 17242321 10.1001/archinte.167.2.188

[5] Vestergaard P, Rejnmark L, Mosekilde L (2006) Anxiolytics, sedatives, antidepressants, neuroleptics and the risk of fracture. Osteoporos Int 17: 807–816. 16520889 10.1007/s00198-005-0065-y

[6] Diem SJ, Blackwell TL, Stone KL, Yaffe K, Haney EM, et al (2007) Use of antidepressants and rates of hip bone loss in older women: the study of osteoporotic fractures. Arch Intern Med 167: 1240–1245. 17592096 10.1001/archinte.167.12.1240

[7] Williams LJ, Henry MJ, Berk M, Dodd S, Jacka FN, et al (2008) Selective serotonin reuptake inhibitor use and bone mineral density in women with a history of depression. Int Clin Psychopharmacol 23: 84–87. 18301122 10.1097/YIC.0b013e3282f2b3bb

[8] Karege F, Widmer J, Bovier P, Gaillard JM (1994) Platelet serotonin and plasma tryptophan in depressed patients: effect of drug treatment and clinical outcome. Neuropsychopharmacology 10: 207–214. 7916918 10.1038/npp.1994.23

[9] Rothman RB, Zolkowska D, Baumann MH (2008) Serotonin (5-HT) transporter ligands affect plasma 5-HT in rats. Ann N Y Acad Sci 1139: 268–284. 18991872 10.1196/annals.1432.042

[10] Zolkowska D, Baumann MH, Rothman RB (2008) Chronic fenfluramine administration increases plasma serotonin (5-hydroxytryptamine) to nontoxic levels. J Pharmacol Exp Ther 324: 791–797. 18032571 10.1124/jpet.107.132654

[11] Cheng S, Lyytikainen A, Kroger H, Lamberg-Allardt C, Alen M, et al (2005) Effects of calcium, dairy product, and vitamin D supplementation on bone mass accrual and body composition in 10-12-y-old girls: a 2-y randomized trial. Am J Clin Nutr 82: 1115–1126 quiz 1147–1118. 16280447 10.1093/ajcn/82.5.1115

[12] Lyytikainen A, Lamberg-Allardt C, Kannas L, Cheng S (2005) Food consumption and nutrient intakes with a special focus on milk product consumption in early pubertal girls in Central Finland. Public Health Nutr 8: 284–289. 15918925 10.1079/phn2004703

[13] Cheng S, Volgyi E, Tylavsky FA, Lyytikainen A, Tormakangas T, et al (2009) Trait-specific tracking and determinants of body composition: a 7-year follow-up study of pubertal growth in girls. BMC Med 7: 5. 19171028 10.1186/1741-7015-7-5 PMC2639618

[14] Kakonen SM, Hellman J, Karp M, Laaksonen P, Obrant KJ, et al (2000) Development and evaluation of three immunofluorometric assays that measure different forms of osteocalcin in serum. Clin Chem 46: 332–337. 10702519

[15] Bliziotes M, Eshleman A, Burt-Pichat B, Zhang XW, Hashimoto J, et al (2006) Serotonin transporter and receptor expression in osteocytic MLO-Y4 cells. Bone 39: 1313–1321. 16884969 10.1016/j.bone.2006.06.009 PMC1766480

[16] Gustafsson BI, Thommesen L, Stunes AK, Tommeras K, Westbroek I, et al (2006) Serotonin and fluoxetine modulate bone cell function in vitro. J Cell Biochem 98: 139–151. 16408289 10.1002/jcb.20734

[17] Westbroek I, van der Plas A, de Rooij KE, Klein-Nulend J, Nijweide PJ (2001) Expression of serotonin receptors in bone. J Biol Chem 276: 28961–28968. 11387323 10.1074/jbc.M101824200

[18] Collet C, Schiltz C, Geoffroy V, Maroteaux L, Launay JM, et al (2008) The serotonin 5-HT2B receptor controls bone mass via osteoblast recruitment and proliferation. FASEB J 22: 418–427. 17846081 10.1096/fj.07-9209com PMC5409955

[19] Warden SJ, Robling AG, Sanders MS, Bliziotes MM, Turner CH (2005) Inhibition of the serotonin (5-hydroxytryptamine) transporter reduces bone accrual during growth. Endocrinology 146: 685–693. 15539550 10.1210/en.2004-1259

[20] Modder UI, Achenbach SJ, Amin S, Riggs BL, Melton LJ, et al (2009) Relation of Serum Serotonin Levels to Bone Density and Structural Parameters in Women. J Bone Miner Res 10.1359/jbmr.090721 PMC3153390 19594297

[21] Blundell JE (1984) Serotonin and appetite. Neuropharmacology 23: 1537–1551. 6152027 10.1016/0028-3908(84)90098-4

[22] Curzon G (1990) Serotonin and appetite. Ann N Y Acad Sci 600: 521–530 discussion 530–521. 2252331 10.1111/j.1749-6632.1990.tb16907.x

[23] Spigset O (1990) The role of serotonin in normal appetite regulation and in the pathogenesis of anorexia/bulimia. Nord Med 105: 292–297. 2251105

[24] Leibowitz SF, Weiss GF, Suh JS (1990) Medial hypothalamic nuclei mediate serotonin's inhibitory effect on feeding behavior. Pharmacol Biochem Behav 37: 735–742. 2093178 10.1016/0091-3057(90)90556-w

[25] Turner CH (2006) Bone strength: current concepts. Ann N Y Acad Sci 1068: 429–446. 16831941 10.1196/annals.1346.039

[26] Currey J (2002) Bone: structure and mechanics. Princeton, NJ: Princeton University Press.

